# Bis(nitrato-κ*O*)bis­[4,4,5,5-tetra­methyl-2-(5-methyl-1*H*-imidazol-4-yl-κ*N* ^3^)-2-imidazoline-1-oxyl 3-oxide-κ*O*]nickel(II)

**DOI:** 10.1107/S1600536811040402

**Published:** 2011-10-08

**Authors:** Jiu Li Chang, Zhi Yong Gao

**Affiliations:** a College of Chemistry and Environmental Science, Henan Normal University, Xinxiang 453002, People’s Republic of China

## Abstract

In the centrosymmetric mononuclear title complex, [Ni(NO_3_)_2_(C_11_H_17_N_4_O_2_)_2_], the Ni^II^ atom displays a distorted octa­hedral coordination geometry and is six-coordinated by two *N*,*O*-bidentate nitronyl nitroxide radical ligands and two monodentate nitrate anions.

## Related literature

For general background to mol­ecular magnetic materials, see: Li *et al.* (2004 ▶); Wang *et al.* (2008 ▶); Yamamoto *et al.* (2001 ▶). For the synthesis, see: Ullman *et al.* (1970 ▶, 1972 ▶). For the related isomorphous Co complex, see: Gao *et al.* (2010 ▶). 
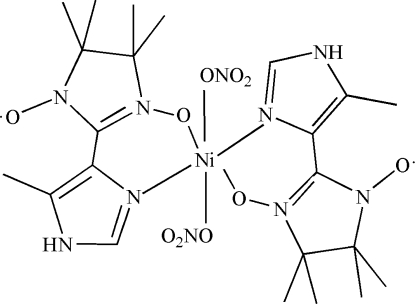


## Experimental

### 

#### Crystal data

[Ni(NO_3_)_2_(C_11_H_17_N_4_O_2_)_2_]
*M* *_r_* = 657.30
Monoclinic, 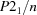

*a* = 7.8313 (5) Å
*b* = 10.7772 (8) Å
*c* = 17.3009 (12) Å
β = 101.464 (1)°
*V* = 1431.07 (17) Å^3^
*Z* = 2
Mo *K*α radiation
μ = 0.75 mm^−1^
*T* = 295 K
0.43 × 0.17 × 0.09 mm

#### Data collection

Bruker SMART CCD area-detector diffractometer
Absorption correction: multi-scan (*SADABS*; Bruker, 2002 ▶) *T*_min_ = 0.741, *T*_max_ = 0.939
12170 measured reflections
3280 independent reflections
2891 reflections with *I* > 2σ(*I*)
*R*_int_ = 0.016

#### Refinement

*R*[*F* ^2^ > 2σ(*F* ^2^)] = 0.029
*wR*(*F* ^2^) = 0.084
*S* = 1.03
3280 reflections
201 parameters
H-atom parameters constrained
Δρ_max_ = 0.54 e Å^−3^
Δρ_min_ = −0.29 e Å^−3^

### 

Data collection: *SMART* (Bruker, 2002 ▶); cell refinement: *SAINT* (Bruker, 2002 ▶); data reduction: *SAINT*; program(s) used to solve structure: *SHELXS97* (Sheldrick, 2008 ▶); program(s) used to refine structure: *SHELXL97* (Sheldrick, 2008 ▶); molecular graphics: *SHELXTL* (Sheldrick, 2008 ▶); software used to prepare material for publication: *publCIF* (Westrip, 2010 ▶).

## Supplementary Material

Crystal structure: contains datablock(s) I, global. DOI: 10.1107/S1600536811040402/bg2419sup1.cif

Structure factors: contains datablock(s) I. DOI: 10.1107/S1600536811040402/bg2419Isup2.hkl

Additional supplementary materials:  crystallographic information; 3D view; checkCIF report

## Figures and Tables

**Fig. 1.**
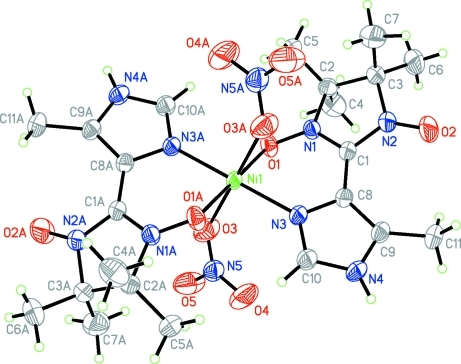
Ellipsoid plot of the title complex with atom labelling. Displacement ellipsoids are drawn at 50% probability level [symmetry code A:-x,-y + 2,-z].

## supplementary crystallographic information

### Comment

The design and synthesis of molecular-based magnetic materials with ferromagnetic ordering is one of the focus in molecular materials research (Yamamoto *et al.*, 2001). In the preparation of molecular magnetic materials,transition metal complexes with organic radical ligands have found widespread interest in recent years (Li *et al.*, 2004; Gao *et al.*, 2010; Wang *et al.*, 2008). A wide variety of transition metal complexes have been prepared with nitronyl nitroxide radical ligands. In this contribution, we report the synthesis and crystal structure of the title Ni^II^ complex Ni(NO_3_)_2_(C_11_H_17_N_4_O_2_)_2_ (I).

An ellipsoid plot of I is shown in Fig.1. The nickel(II) ion presents a distorted octahedral coordination environment, and it is coordinated by two monodentate nitrate anions and two chelating nitronyl nitroxide radicals which lead two six-membered rings.

The complex is very similar to a recently published one (Gao *et al.*, 2010) where the nitrate anions in (I) are replaced by methanol molecules, and charge balance is achieved via two non bonded perchlorate counteranions.

### Experimental

The nitronyl nitroxide radical, 4,4,5,5-tetramethyl-2-(5-methylimidazol-4-yl)-2-imidazoline-1-oxyl-3-oxide, was prepared according to the literature method (Ullman *et al.* 1970; Ullman *et al.* 1972). The title complex [Ni(NO_3_)_2_(C_11_H_17_N_4_O_2_)_2_] was synthesized by adding Ni(NO_3_)_2_.6H_2_O (0.25 mmol) to 25 ml of an ethanol solution containing the nitroxide radical ligands(0.50 mmol). The mixture was stirred for 3 h at room temperature and then filtered off. The blue filtrate was allowed to stand at room temperature and dark blue crystals suitable for X-ray analysis were obtained after two weeks.

### Refinement

All H atoms attached to C and N atom were posiitoned geometrically and treated as riding with C—H = 0.93 Å(methine) or 0.96 Å(methyl),*N*—H = 0.86Å. Those pertaining to methyl groups were allowed to rotate as well. Displacement factors were taken as *U*_iso_(H) = 1.2Ueq(Cmethine) or *U*_iso_(H) = 1.5Ueq(Cmethyl).

### Crystal data

**Table:**

[Ni(NO_3_)_2_(C_11_H_17_N_4_O_2_)_2_]	*F*(000) = 688
*M**_r_* = 657.30	*D*_x_ = 1.525 Mg m^−^^3^
Monoclinic, *P*2_1_/*n*	Mo *K*α radiation, λ = 0.71073 Å
Hall symbol: -P 2yn	Cell parameters from 4292 reflections
*a* = 7.8313 (5) Å	θ = 2.7–28.3°
*b* = 10.7772 (8) Å	µ = 0.75 mm^−^^1^
*c* = 17.3009 (12) Å	*T* = 295 K
β = 101.464 (1)°	Block, dark blue
*V* = 1431.07 (17) Å^3^	0.43 × 0.17 × 0.09 mm
*Z* = 2	

### Data collection

**Table:**

Bruker SMART CCD area-detector diffractometer	3280 independent reflections
Radiation source: fine-focus sealed tube	2891 reflections with *I* > 2σ(*I*)
graphite	*R*_int_ = 0.016
φ and ω scans	θ_max_ = 27.5°, θ_min_ = 2.7°
Absorption correction: multi-scan (*SADABS*; Bruker, 2002)	*h* = −10→10
*T*_min_ = 0.741, *T*_max_ = 0.939	*k* = −13→14
12170 measured reflections	*l* = −22→22

### Refinement

**Table:**

Refinement on *F*^2^	Primary atom site location: structure-invariant direct methods
Least-squares matrix: full	Secondary atom site location: difference Fourier map
*R*[*F*^2^ > 2σ(*F*^2^)] = 0.029	Hydrogen site location: inferred from neighbouring sites
*wR*(*F*^2^) = 0.084	H-atom parameters constrained
*S* = 1.03	*w* = 1/[σ^2^(*F*_o_^2^) + (0.047*P*)^2^ + 0.4906*P*] where *P* = (*F*_o_^2^ + 2*F*_c_^2^)/3
3280 reflections	(Δ/σ)_max_ < 0.001
201 parameters	Δρ_max_ = 0.54 e Å^−^^3^
0 restraints	Δρ_min_ = −0.29 e Å^−^^3^

### Special details

**Table:**

Geometry. All esds (except the esd in the dihedral angle between two l.s. planes) are estimated using the full covariance matrix. The cell esds are taken into account individually in the estimation of esds in distances, angles and torsion angles; correlations between esds in cell parameters are only used when they are defined by crystal symmetry. An approximate (isotropic) treatment of cell esds is used for estimating esds involving l.s. planes.
Refinement. Refinement of *F*^2^ against ALL reflections. The weighted *R*-factor *wR* and goodness of fit *S* are based on *F*^2^, conventional *R*-factors *R* are based on *F*, with *F* set to zero for negative *F*^2^. The threshold expression of *F*^2^ > σ(*F*^2^) is used only for calculating *R*-factors(gt) *etc*. and is not relevant to the choice of reflections for refinement. *R*-factors based on *F*^2^ are statistically about twice as large as those based on *F*, and *R*- factors based on ALL data will be even larger.

### Fractional atomic coordinates and isotropic or equivalent isotropic displacement parameters (Å^2^)

**Table:**

	*x*	*y*	*z*	*U*_iso_*/*U*_eq_	
Ni1	0.0000	1.0000	0.0000	0.02551 (9)	
O1	0.00030 (13)	0.87872 (10)	0.09062 (7)	0.0356 (3)	
O2	0.56556 (16)	0.72951 (13)	0.14208 (11)	0.0628 (4)	
O3	−0.11891 (17)	1.14165 (13)	0.06167 (9)	0.0535 (4)	
O4	0.0728 (2)	1.27774 (17)	0.11237 (13)	0.0793 (5)	
O5	−0.1945 (2)	1.29491 (15)	0.12542 (11)	0.0695 (5)	
N1	0.13496 (15)	0.80628 (12)	0.11228 (7)	0.0293 (3)	
N2	0.40216 (17)	0.73607 (13)	0.13945 (9)	0.0389 (3)	
N3	0.25299 (15)	1.03606 (12)	0.04597 (7)	0.0288 (3)	
N4	0.50665 (16)	1.12494 (12)	0.08397 (8)	0.0341 (3)	
H4D	0.5873	1.1801	0.0877	0.041*	
N5	−0.07686 (18)	1.23919 (14)	0.10055 (9)	0.0409 (3)	
C1	0.30161 (18)	0.83775 (14)	0.11699 (9)	0.0293 (3)	
C2	0.1134 (2)	0.68040 (14)	0.14566 (10)	0.0335 (3)	
C3	0.2948 (2)	0.62185 (15)	0.14399 (10)	0.0378 (4)	
C4	0.0799 (3)	0.70293 (19)	0.22840 (12)	0.0527 (5)	
H4A	−0.0233	0.7523	0.2252	0.079*	
H4B	0.1775	0.7459	0.2593	0.079*	
H4C	0.0644	0.6248	0.2528	0.079*	
C5	−0.0410 (2)	0.61400 (18)	0.09556 (13)	0.0506 (5)	
H5A	−0.1470	0.6545	0.1013	0.076*	
H5B	−0.0432	0.5293	0.1125	0.076*	
H5C	−0.0305	0.6162	0.0412	0.076*	
C6	0.3723 (3)	0.5458 (2)	0.21666 (14)	0.0598 (6)	
H6A	0.4829	0.5128	0.2109	0.090*	
H6B	0.2949	0.4789	0.2224	0.090*	
H6C	0.3880	0.5979	0.2626	0.090*	
C7	0.3019 (3)	0.5479 (2)	0.06956 (14)	0.0571 (5)	
H7A	0.4210	0.5298	0.0677	0.086*	
H7B	0.2505	0.5957	0.0240	0.086*	
H7C	0.2386	0.4717	0.0701	0.086*	
C8	0.36190 (18)	0.95783 (14)	0.09802 (9)	0.0276 (3)	
C9	0.52150 (19)	1.01413 (14)	0.12285 (9)	0.0299 (3)	
C10	0.34633 (19)	1.13445 (15)	0.03893 (10)	0.0337 (3)	
H10A	0.3070	1.2021	0.0070	0.040*	
C11	0.6814 (2)	0.98292 (17)	0.18240 (11)	0.0404 (4)	
H11C	0.7305	1.0575	0.2080	0.061*	
H11D	0.7650	0.9439	0.1566	0.061*	
H11A	0.6515	0.9273	0.2210	0.061*	

### Atomic displacement parameters (Å^2^)

**Table:**

	*U*^11^	*U*^22^	*U*^33^	*U*^12^	*U*^13^	*U*^23^
Ni1	0.01760 (14)	0.02601 (15)	0.03174 (15)	−0.00258 (9)	0.00206 (10)	0.00404 (10)
O1	0.0231 (5)	0.0370 (6)	0.0476 (6)	0.0047 (4)	0.0095 (5)	0.0158 (5)
O2	0.0250 (6)	0.0460 (8)	0.1147 (13)	0.0068 (5)	0.0077 (7)	0.0061 (8)
O3	0.0379 (7)	0.0565 (8)	0.0675 (9)	−0.0097 (6)	0.0138 (6)	−0.0187 (7)
O4	0.0414 (8)	0.0697 (11)	0.1226 (15)	−0.0215 (8)	0.0061 (9)	−0.0141 (10)
O5	0.0641 (10)	0.0521 (9)	0.0981 (13)	−0.0120 (7)	0.0301 (9)	−0.0232 (8)
N1	0.0246 (6)	0.0287 (6)	0.0341 (6)	0.0005 (5)	0.0043 (5)	0.0069 (5)
N2	0.0255 (6)	0.0314 (7)	0.0570 (9)	0.0015 (5)	0.0010 (6)	0.0022 (6)
N3	0.0213 (6)	0.0293 (6)	0.0349 (7)	−0.0026 (5)	0.0038 (5)	0.0022 (5)
N4	0.0240 (6)	0.0355 (7)	0.0426 (7)	−0.0093 (5)	0.0065 (5)	0.0005 (6)
N5	0.0339 (7)	0.0410 (8)	0.0465 (8)	−0.0070 (6)	0.0048 (6)	0.0088 (6)
C1	0.0228 (7)	0.0301 (7)	0.0335 (7)	−0.0003 (5)	0.0022 (5)	0.0008 (6)
C2	0.0318 (8)	0.0281 (7)	0.0389 (8)	−0.0039 (6)	0.0026 (6)	0.0081 (6)
C3	0.0334 (8)	0.0277 (7)	0.0481 (9)	0.0001 (6)	−0.0021 (7)	0.0029 (7)
C4	0.0653 (13)	0.0497 (11)	0.0470 (10)	0.0018 (9)	0.0204 (9)	0.0140 (8)
C5	0.0362 (9)	0.0387 (9)	0.0703 (13)	−0.0094 (7)	−0.0052 (8)	0.0032 (9)
C6	0.0520 (12)	0.0473 (11)	0.0704 (14)	0.0064 (9)	−0.0113 (10)	0.0195 (10)
C7	0.0538 (12)	0.0469 (11)	0.0690 (14)	0.0022 (9)	0.0082 (10)	−0.0133 (10)
C8	0.0219 (7)	0.0289 (7)	0.0319 (7)	0.0002 (5)	0.0048 (5)	−0.0008 (6)
C9	0.0226 (7)	0.0356 (8)	0.0320 (7)	−0.0013 (6)	0.0064 (6)	−0.0031 (6)
C10	0.0265 (7)	0.0334 (8)	0.0408 (8)	−0.0039 (6)	0.0060 (6)	0.0049 (6)
C11	0.0240 (8)	0.0500 (10)	0.0443 (9)	−0.0005 (7)	−0.0004 (7)	−0.0003 (7)

### Geometric parameters (Å, °)

**Table:**

Ni1—N3^i^	2.0206 (12)	C2—C4	1.525 (3)
Ni1—N3	2.0206 (12)	C2—C3	1.560 (2)
Ni1—O1^i^	2.0408 (10)	C3—C7	1.525 (3)
Ni1—O1	2.0408 (10)	C3—C6	1.522 (3)
Ni1—O3	2.1751 (13)	C4—H4A	0.9600
Ni1—O3^i^	2.1751 (13)	C4—H4B	0.9600
O1—N1	1.3057 (16)	C4—H4C	0.9600
O2—N2	1.2733 (18)	C5—H5A	0.9600
O3—N5	1.256 (2)	C5—H5B	0.9600
O4—N5	1.221 (2)	C5—H5C	0.9600
O5—N5	1.246 (2)	C6—H6A	0.9600
N1—C1	1.3351 (18)	C6—H6B	0.9600
N1—C2	1.4971 (19)	C6—H6C	0.9600
N2—C1	1.3603 (19)	C7—H7A	0.9600
N2—C3	1.501 (2)	C7—H7B	0.9600
N3—C10	1.3073 (19)	C7—H7C	0.9600
N3—C8	1.3942 (19)	C8—C9	1.379 (2)
N4—C10	1.344 (2)	C9—C11	1.493 (2)
N4—C9	1.364 (2)	C10—H10A	0.9300
N4—H4D	0.8600	C11—H11C	0.9600
C1—C8	1.438 (2)	C11—H11D	0.9600
C2—C5	1.520 (2)	C11—H11A	0.9600
			
N3^i^—Ni1—N3	180.0	C7—C3—C6	109.94 (17)
N3^i^—Ni1—O1^i^	88.25 (5)	N2—C3—C2	101.00 (12)
N3—Ni1—O1^i^	91.75 (5)	C7—C3—C2	114.39 (14)
N3^i^—Ni1—O1	91.75 (5)	C6—C3—C2	114.66 (16)
N3—Ni1—O1	88.25 (5)	C2—C4—H4A	109.5
O1^i^—Ni1—O1	180.00 (4)	C2—C4—H4B	109.5
N3^i^—Ni1—O3	81.15 (5)	H4A—C4—H4B	109.5
N3—Ni1—O3	98.85 (5)	C2—C4—H4C	109.5
O1^i^—Ni1—O3	89.55 (5)	H4A—C4—H4C	109.5
O1—Ni1—O3	90.45 (5)	H4B—C4—H4C	109.5
N3^i^—Ni1—O3^i^	98.85 (5)	C2—C5—H5A	109.5
N3—Ni1—O3^i^	81.15 (5)	C2—C5—H5B	109.5
O1^i^—Ni1—O3^i^	90.45 (5)	H5A—C5—H5B	109.5
O1—Ni1—O3^i^	89.55 (5)	C2—C5—H5C	109.5
O3—Ni1—O3^i^	180.00 (6)	H5A—C5—H5C	109.5
N1—O1—Ni1	118.74 (8)	H5B—C5—H5C	109.5
N5—O3—Ni1	139.27 (11)	C3—C6—H6A	109.5
O1—N1—C1	126.13 (13)	C3—C6—H6B	109.5
O1—N1—C2	120.40 (11)	H6A—C6—H6B	109.5
C1—N1—C2	112.98 (12)	C3—C6—H6C	109.5
O2—N2—C1	125.14 (14)	H6A—C6—H6C	109.5
O2—N2—C3	121.44 (13)	H6B—C6—H6C	109.5
C1—N2—C3	112.16 (12)	C3—C7—H7A	109.5
C10—N3—C8	105.61 (12)	C3—C7—H7B	109.5
C10—N3—Ni1	129.94 (11)	H7A—C7—H7B	109.5
C8—N3—Ni1	124.33 (10)	C3—C7—H7C	109.5
C10—N4—C9	109.33 (13)	H7A—C7—H7C	109.5
C10—N4—H4D	125.3	H7B—C7—H7C	109.5
C9—N4—H4D	125.3	C9—C8—N3	109.59 (13)
O4—N5—O5	121.93 (17)	C9—C8—C1	130.05 (14)
O4—N5—O3	120.99 (17)	N3—C8—C1	120.35 (13)
O5—N5—O3	117.07 (15)	N4—C9—C8	104.39 (13)
N1—C1—N2	108.43 (13)	N4—C9—C11	121.00 (14)
N1—C1—C8	125.18 (13)	C8—C9—C11	134.40 (15)
N2—C1—C8	126.34 (13)	N3—C10—N4	111.07 (14)
N1—C2—C5	110.16 (13)	N3—C10—H10A	124.5
N1—C2—C4	105.75 (14)	N4—C10—H10A	124.5
C5—C2—C4	110.14 (16)	C9—C11—H11C	109.5
N1—C2—C3	100.75 (12)	C9—C11—H11D	109.5
C5—C2—C3	115.06 (15)	H11C—C11—H11D	109.5
C4—C2—C3	114.15 (14)	C9—C11—H11A	109.5
N2—C3—C7	105.64 (15)	H11C—C11—H11A	109.5
N2—C3—C6	110.44 (15)	H11D—C11—H11A	109.5

Symmetry codes: (i) −*x*, −*y*+2, −*z*.
